# How I do it: intracranially extended C2 schwannoma resection with tailored craniectomy

**DOI:** 10.1007/s00701-026-06785-8

**Published:** 2026-05-07

**Authors:** Lorenzo Gelmini, Amedeo Piazza, Marco Maria Fontanella, Edoardo Agosti

**Affiliations:** 1 Neurosurgery, Department of Medical and Surgical Specialties, Radiological Sciences and Public Health, University of Brescia, Brescia, Italy; 2 Division of Neurosurgery, Department of Neuroscience, Sapienza University of Rome, Via Enrico Viarisio 2, 00139 Rome, Italy

**Keywords:** Tailored craniectomy, Hemilaminectomy, Vertebral artery, Craniocervical junction, C2 schwannoma

## Abstract

**Background:**

C2 schwannomas are rare neoplasms presenting with variable extensions. Their possible intracranial involvement and close relationship with the V3 segment of the vertebral artery (VA) require a personalized and delicate surgical approach.

**Method:**

We describe the resection steps of an extradural C2 schwannoma with intracranial extension. Indications, advantages, and approach-specific complications are discussed. The main surgical steps are illustrated in an operative video.

**Conclusion:**

A tailored craniectomy adapted to the lesion intracranial extension enables a safe and effective resection of selected extradural, multicompartmental, cranially extended C2 schwannomas.

**Supplementary Information:**

The online version contains supplementary material available at 10.1007/s00701-026-06785-8.

## Introduction

Cervical spinal schwannomas are rare WHO grade 1 tumors arising from the nerve sheaths of cervical roots and occurring across a wide age range, without a significant sex-related difference in incidence [5]. According to Eden’s classification, spinal schwannomas are divided into four types [2, 4]. We present a case of extradural type 3 C2 schwannoma with intracranial extension.

## Relevant surgical anatomy

The craniocervical junction (CCJ) is a complex transitional region composed of three bony elements: the occipital bone, atlas (C1), and axis (C2); and two major joints: the atlanto-occipital and atlanto-axial joints, stabilized by the craniocervical ligaments. Critical neurovascular structures traverse this area, including the upper cervical spinal cord, which continues as the medulla oblongata through the foramen magnum (FM), as well as the lower cranial nerves and the C1 and C2 roots and ganglia.

**Fig. 1:**
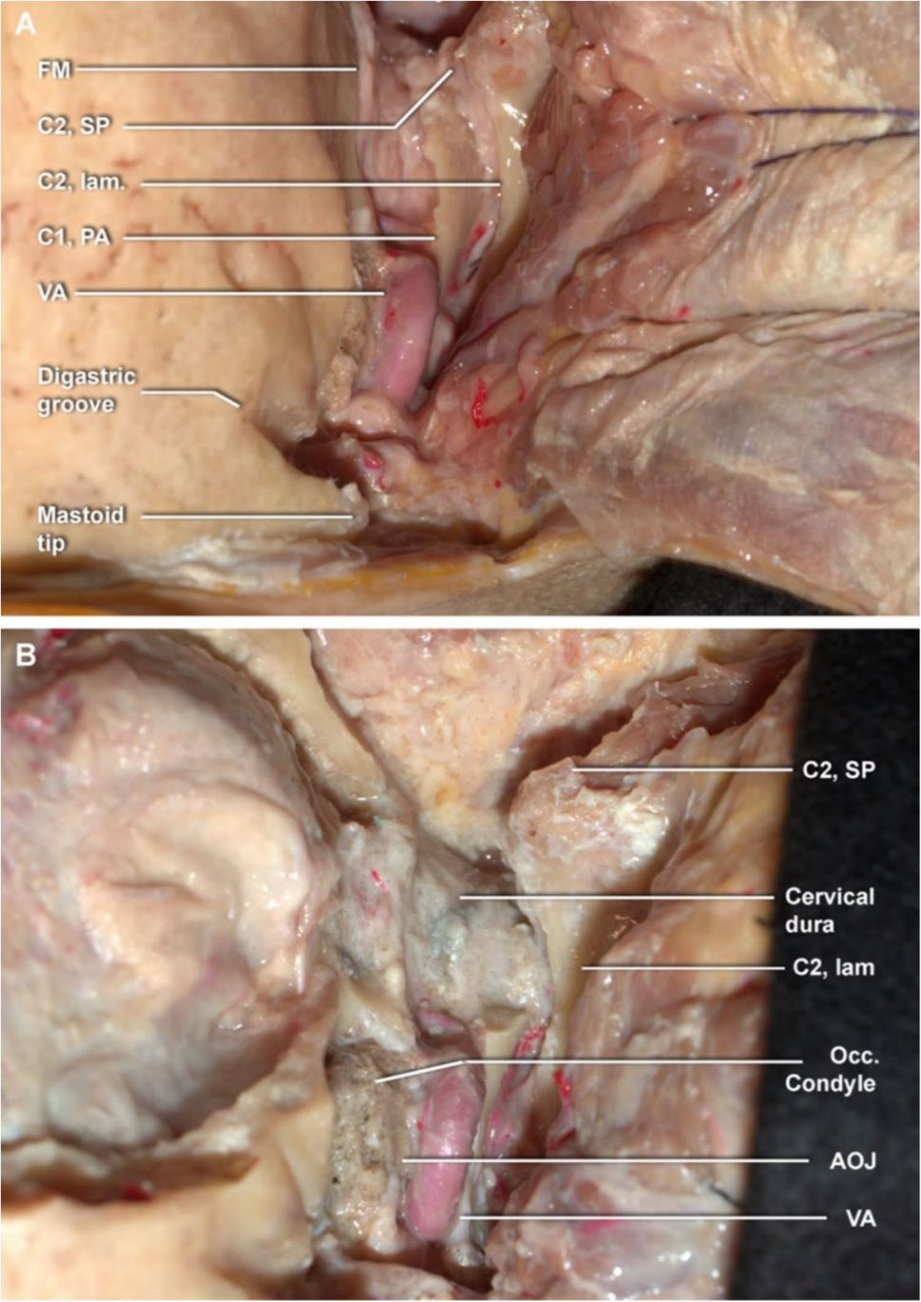
Anatomical dissection performed at our institution showing an overview of the upper cervical spine and craniocervical junction anatomy after a left far-lateral approach. **A** Before C1 hemilaminectomy. **B** After C1 hemilaminectomy. *Abbreviations*: AOJ = Atlanto-occipital joint; FM = Foramen Magnum; lam. = lamina; occ. = occipital; PA = Posterior Arch; SP = Spinous Process; VA = Vertebral artery

From a vascular perspective, the vertebral artery (VA), particularly its V3 segment, is the main structure of concern. It courses within the suboccipital triangle, bounded by the rectus capitis posterior major and the obliquus capitis inferior (forming the inferomedial apex) and the obliquus capitis superior (forming the superolateral base) [3] (Fig. 1).

## Description of the technique

A 64 year-old female presented to our department with a three-year history of burning dysesthesias in the hands and progressively worsening tingling paresthesia, associated with marked asthenia and headache with nausea in the previous weeks. Neurological examination demonstrated a positive left Hoffmann sign and hyperexcitability of the left biceps reflex.

**Fig. 2:**
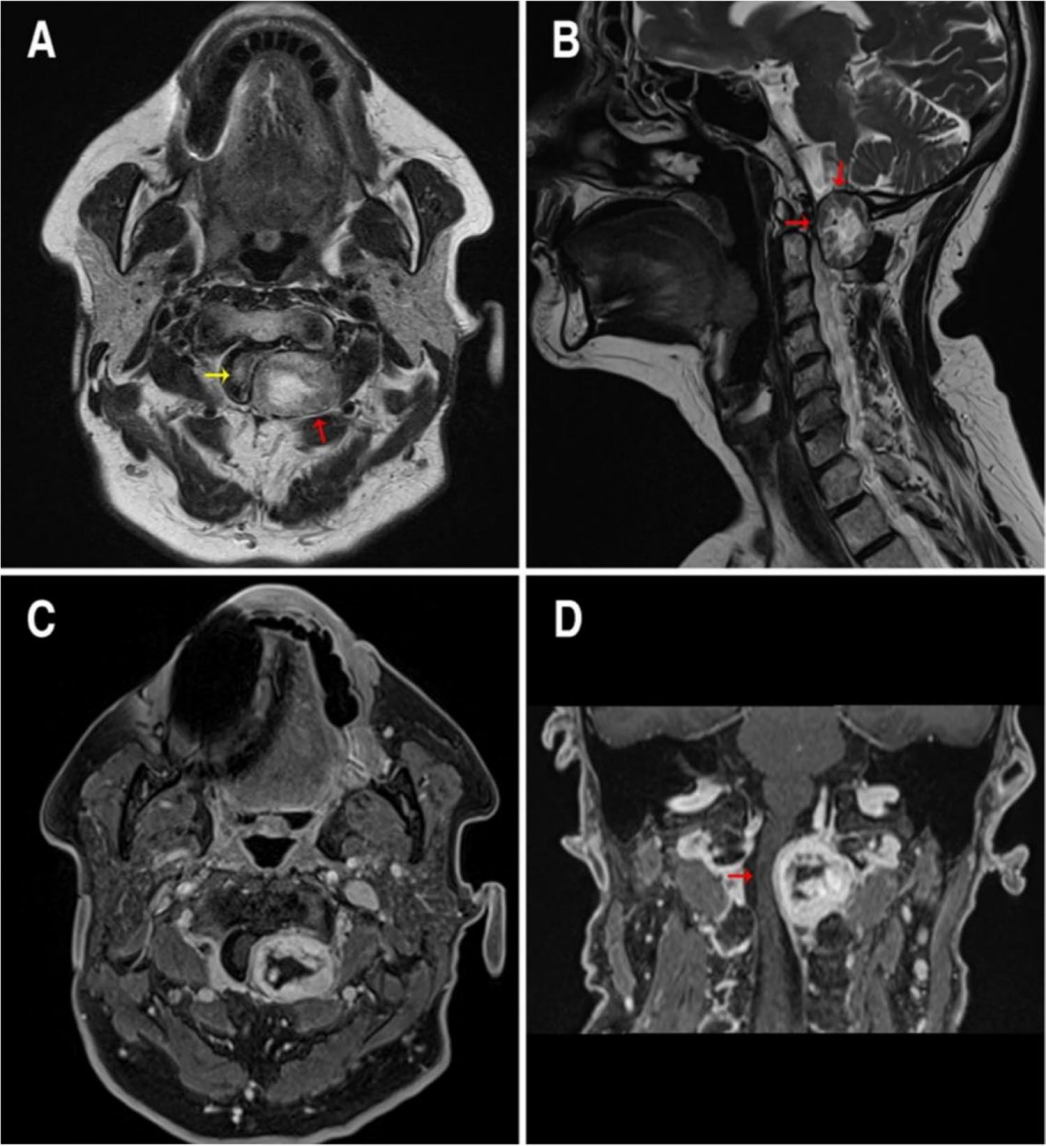
MRI images showing a left cervical extra-axial expansive lesion (22 × 28 × 32 mm) with intra- and extracanalicular extension at the C1–C2 level and occipital extension through the foramen magnum (red arrows). **A** Axial T2-weighted MRI view highlighting the intracanalicular extension of the lesion and altered signal in the cervical spinal cord, indicating compressive myelopathy (yellow arrow). **B** Sagittal T2-weighted MRI view showing the tumor’s intracranial extension. **C** Axial T1-weighted MRI view demonstrating the encapsulated nature of the tumor. **D** Coronal MRI reconstruction showing spinal cord compression and rightward displacement

Magnetic resonance imaging (MRI) revealed a left cervical extra-axial expansive lesion (22 × 28 × 32 mm) with intra- and extracanalar extension at the C1–C2 level and a superior intracranial extension toward the FM (Fig. 2A, B and C). The lesion caused marked compression and rightward displacement of the upper cervical spinal cord, with signs of compressive myelopathy (Fig. 2A and D). Considering the debilitating symptoms and the radiological findings, surgical removal of the lesion was planned.

**Fig. 3:**
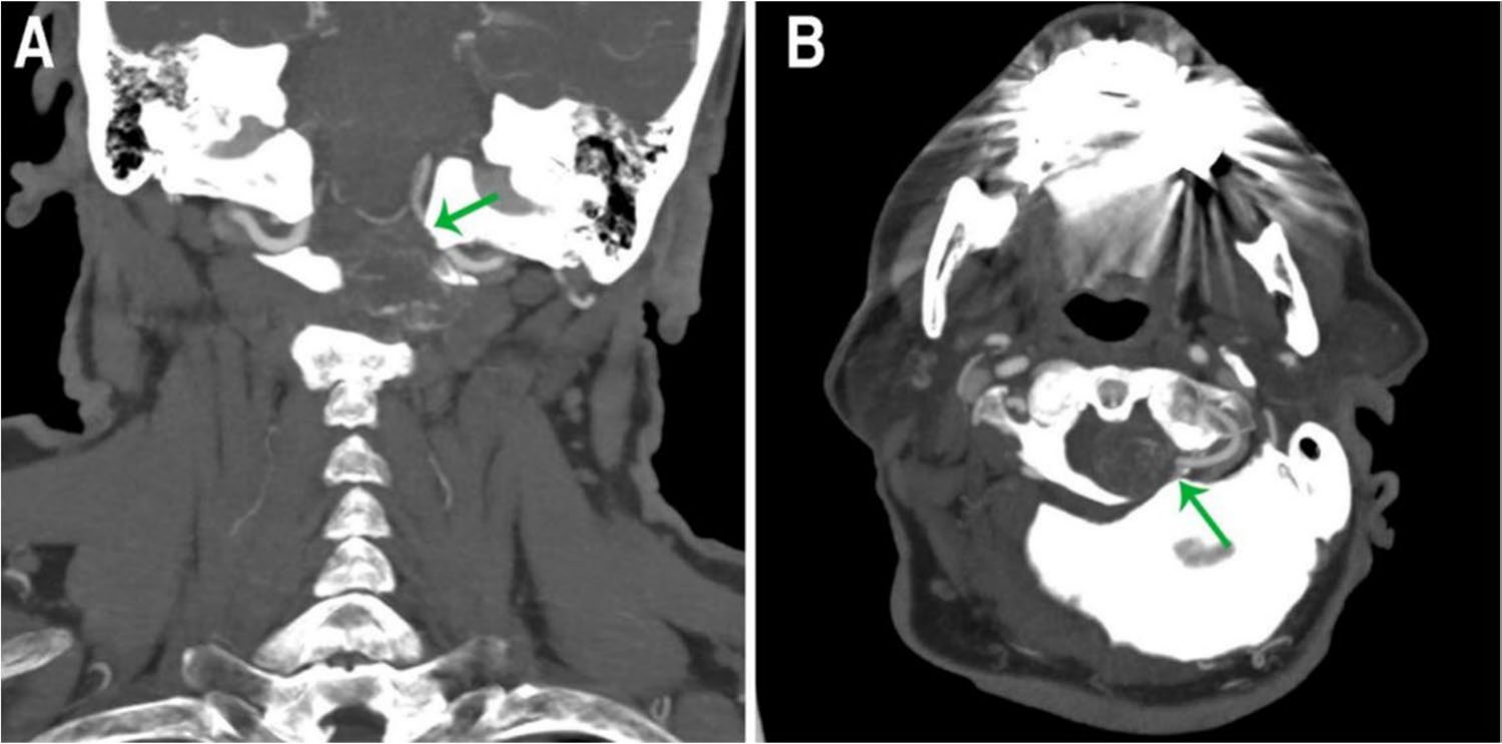
CTA views of the lesion demonstrating its close relationship with the V3 segment of the vertebral artery (green arrows). **A** Coronal view. **B** Axial view

To complete the preoperative assessment, a Computed Tomography Angiography (CTA) was performed, showing that the VA V3 segment coursed at the superolateral margin of the lesion (Fig. 3).

### Positioning

**Fig. 4:**
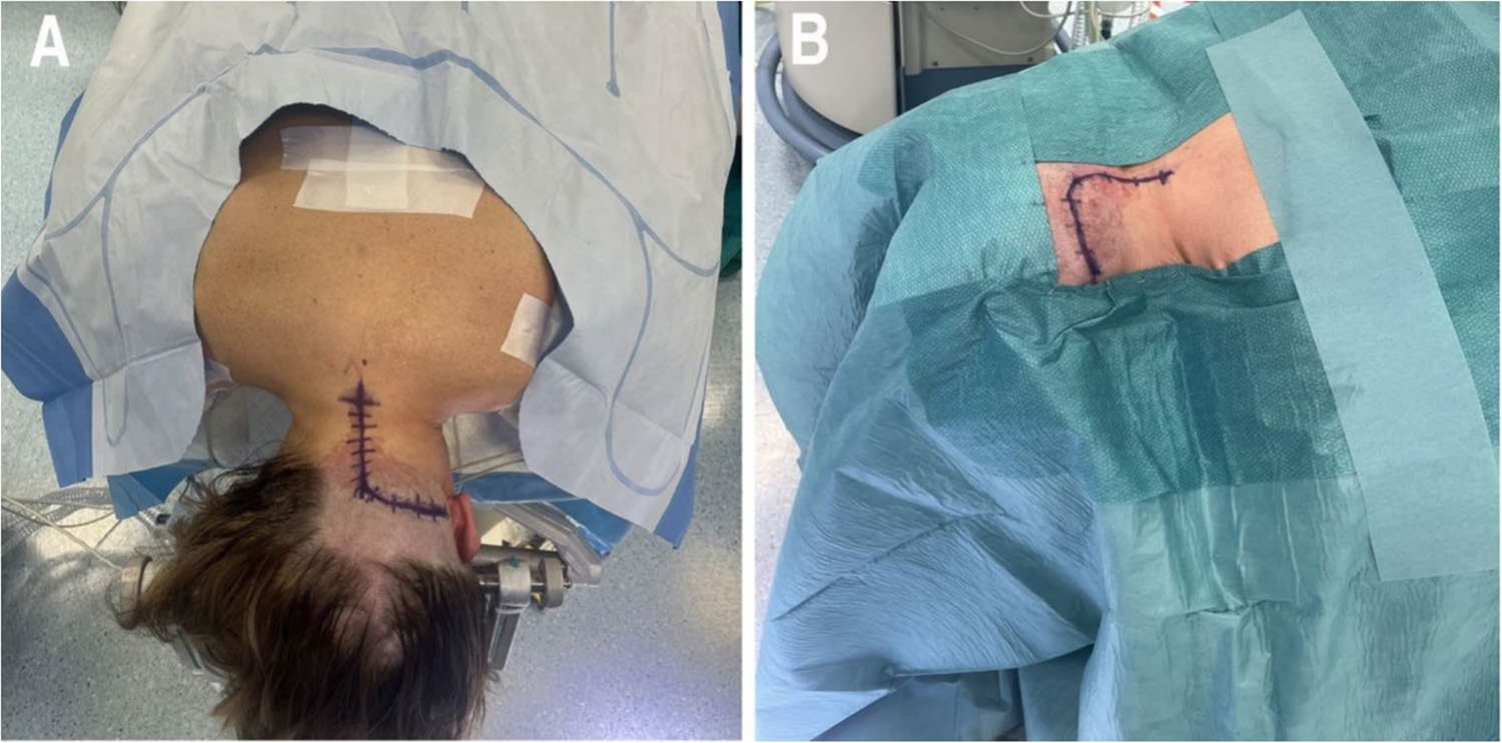
**A** patient in prone position, Hockey-stick skin incisions. **B** surgical field after draoing

The patient was placed in the prone position with the head in a neutral position on the operating table. Intraoperative setting included neuromonitoring (SEPs and MEPs) and an ultrasound Doppler probe (Fig. 4).

### Skin incision

**Fig. 5:**
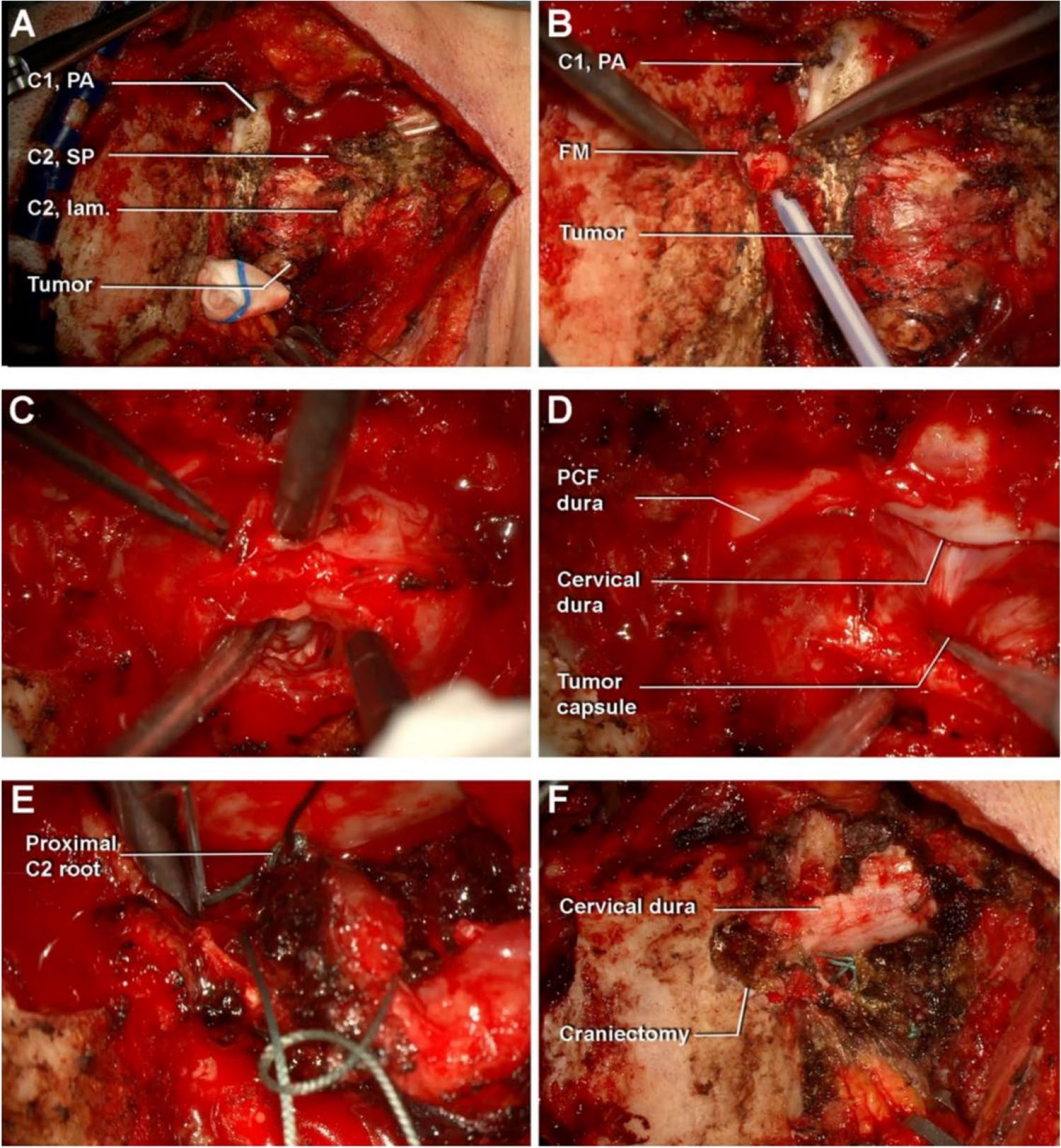
Intraoperative images illustrating key surgical steps. **A** Overview of the surgical anatomy. **B** Verification of the V3 segment of the vertebral artery using an ultrasound Doppler probe. **C** Tumor removal with an ultrasonic aspirator. **D** Tumor capsule dissection — superomedially from the posterior cranial fossa’s dura and medially from the cervical dura. **E** Proximal root ligation with a double knot before sectioning. **F** Final overview of the surgical field showing the lateral suboccipital tailored craniectomy and the cervical spinal cord after C1 hemilaminectomy, C2 partial laminectomy, and tumor removal. *Abbreviations*: FM = Foramen Magnum; PA = Posterior Arch; PCF = Posterior cranial fossa; SP = Spinous Process; VA = Vertebral artery

A left hockey-stick incision was made. Starting from the mastoid tip, the incision was directed superiorly toward the superior nuchal line, then curved medially and extended to the inion. Finally, it was directed inferiorly to reach the third cervical vertebra (C3) (Fig. 4). Before transposing the myocutaneous flap inferolaterally, the suboccipital triangle muscles and the V3 segment of the VA were identified using an interfascial dissection technique. The lateral suboccipital region and the CCJ where then exposed (Fig. 5A). Progressive skeletonization of the C1 posterior tubercle and the C2–C3 spinous processes was performed along the midline avascular plane in a superior-to-inferior direction. The left C1 posterior arch and C2 lamina were then also skeletonized. A large encapsulated lesion was identified between the C1 and C2 foramina, resulting in significant spinal cord compression (Fig. 5A).

### Tumor exposure

The atlanto-occipital membrane was incised and opened. Using an ultrasound Doppler probe, the V3 segment of the VA was identified (Fig. 5B). A left paramedian suboccipital craniectomy was performed by drilling the posterolateral aspect of the FM according to the lesion’s intracranial extension, to expose the tumor’s superior margin and improve surgical maneuverability for subsequent tumor resection. A left C1 hemilaminectomy and partial left C2 laminectomy were then performed using Kerrison and Leksell rongeurs, to complete intracanalar tumor exposure without causing vertebral instability.

### Tumor dissection and removal

Following a second VA check, dissection of the tumor capsule from surrounding tissues was initiated using Penfield dissectors, bipolar forceps, and microscissors. The capsule appeared grey, with a firm–elastic consistency. After incision of the capsule, tumor debulking was performed with an ultrasonic aspirator and Yasargil forceps.

From this point, tumor piecemeal removal and progressive capsule dissection and coagulation were alternated, with frequent VA ultrasound checks (Fig. 5C).

An optimal cleavage plane was identified. The medial surface of the capsule was first dissected completely from the C1–C2 segment of the cervical dura, then superomedially from the posterior cranial fossa dura, and finally superolaterally from the V3 segment of the VA and laterally from the left C1 lateral mass. Lastly, the inferior capsule margin was dissected from the remaining C2 lamina (Fig. 5D).

After complete tumor debulking, inspection inside the capsule excluded tumor intradural extension and identified the root entry point of the lesion. Lateral mobilization of the capsule enabled identification of the proximal C2 root exit from the spinal cord and the distal C2 root exit from the capsule’s lateral margin. After clear visualization, the proximal and distal C2 roots were each ligated with two knots and divided from the tumor proximally and distally, allowing complete removal of the tumor capsule and preventing recurrence (Fig. 5E). Hemostasis was secured using bipolar coagulation and hemostatic foam (Fig. 5F).

**Fig. 6:**
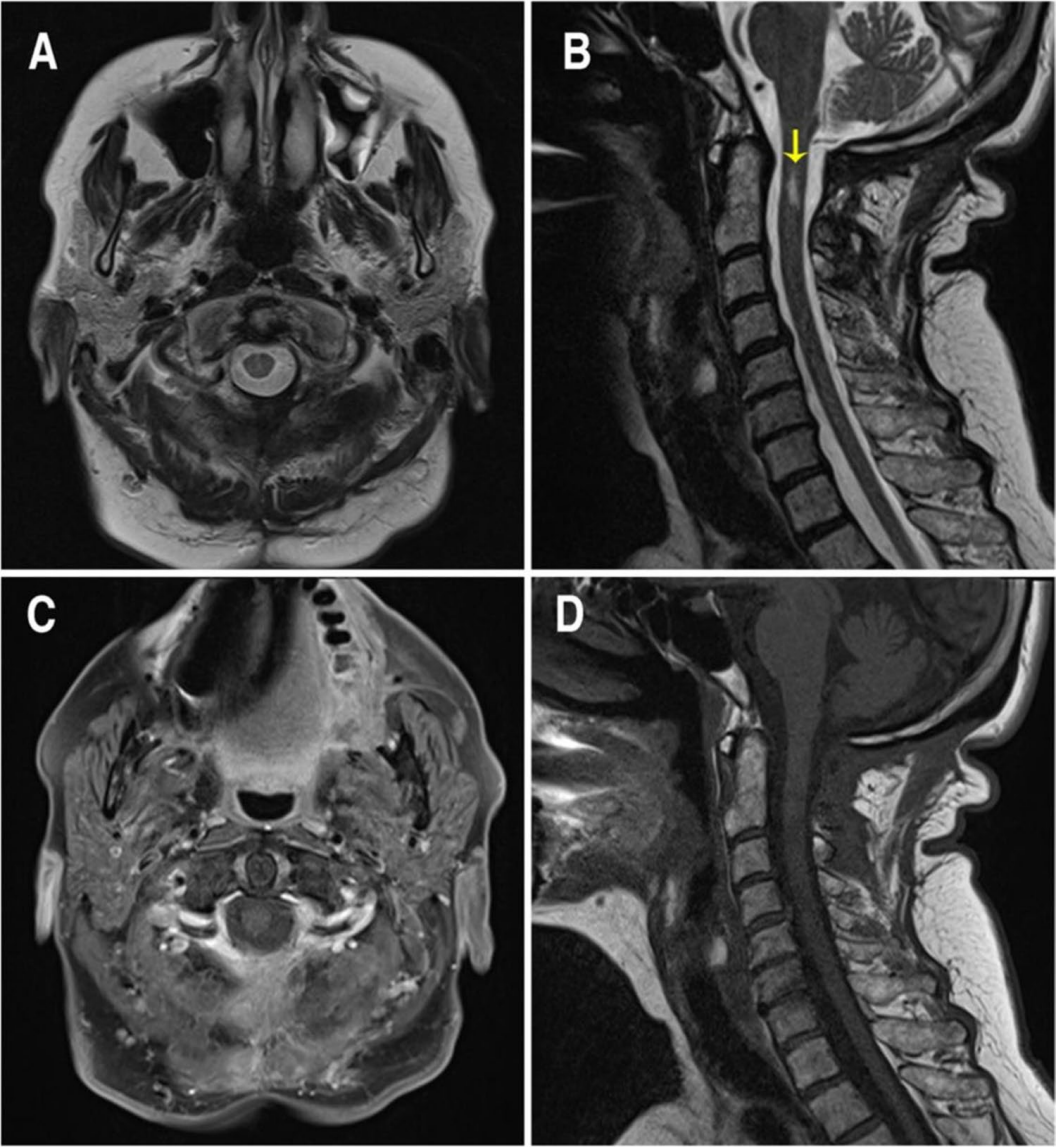
Three-month postoperative MRI. **A** Coronal T2-weighted MRI view showing absence of tumor recurrence and spinal cord decompression. **B** Sagittal T1-weighted MRI view showing signal alteration at the C1–C2 level of the spinal cord, consistent with persistent myelopathy (yellow arrow). **C** Coronal T1-weighted MRI view. **D** Sagittal T1-weighted MRI view showing signs of C1 hemilaminectomy and C2 partial laminectomy

The postoperative course was uneventful, and the patient was discharged on POD 5. Histopathological analysis confirmed WHO grade 1 schwannoma. The 3-month follow-up MRI confirmed GTR of the lesion; however, signs of myelopathy in the cervical spinal cord were still visible (Fig. 6).

On follow-up evaluation, the patient reported relief of preoperative symptoms, except for mild recrudescence of hand paresthesias, consistent with the radiological findings.

## Indications

- Surgical resection is usually recommended in one of these cases: symptomatic patients, patients with spinal cord compression with or without myelopathy, or evidence of tumor growth on imaging
- Tailored craniectomy is recommended for CCJ tumors with intracranial extension and close to the VA V3 segment to obtain tumor exposure and surgical maneuverability

## Limitations

- For lesions without significant lateral or superior extension, a posterior midline approach is preferable [1]
- Radiosurgery should be indicated for recurrent or post-surgical remnant lesion
- If occipito-cervical stabilization is needed, the suboccipital craniectomy extension should be tailored to occipital plaque location

## How to avoid complications

- Preoperative CTA or MRA (Magnetic Resonance Angiography) are important to evaluate the relationship between the tumor and the VA
- In cases of VA encasement, DSA (Digital Subtraction Angiography) and VA embolization are recommended if VA is not dominant
- Intraoperative neuromonitoring to avoid neurological deficits
- Doppler ultrasound to check VA position and prevent VA injury proximally the to embolized segment
- Double-knot proximal root nerve ligation to prevent spinal CSF leakage

## Specific perioperative considerations

A CTA or MRA are essential to plan a safe surgical approach. In the postoperative period, a CT scan is performed, and the patient undergoes regular neurosurgical follow-ups for clinical evaluation. The first follow-up MRI is performed at 3 months, with annual imaging thereafter if no recurrence is detected.

## Specific information for the patient

- Risk of intraoperative life-threatening bleeding, particularly from VA injury, and potential need for blood transfusion
- Risk of postoperative Arnold’s neuralgia and other possible neurological deficits
- In case of preoperative radiological and/or clinical signs of myelopathy, radiological and/or clinical persistence of postoperative myelopathy is possible

## Ten key points

1. A CTA or MRA before surgery is important to assess the relationship with the VA
2. Detailed anatomical knowledge of the V3 segment of the vertebral artery is essential to prevent complications.
3. C1 and C2 skeletonization is performed following the midline avascular plane
4. A tailored craniectomy performed according to tumor extension allows full exposure and improves surgical maneuverability
5. C1 hemilaminectomy and partial C2 laminectomy enable intracanalar tumor exposure
6. Intraoperative Doppler ultrasound helps to avoid VA injury
7. Intraoperative neuromonitoring helps to prevent neurological deficits
8. Alternating capsule dissection, coagulation, and piecemeal tumor removal allows complete separation of the capsule from the surrounding tissues
9. Double-knot proximal root ligation prevents spinal CSF leakage.
10. Root cutting prevents tumor recurrence.

## Supplementary Information

Below is the link to the electronic supplementary material.

ESM 1 Supplementary Material 1 (MP4 453 MB)

## Data Availability

No datasets were generated or analysed during the current study.

## References

[1] Aldea S, Alkhairy A, Joitescu I, Guerinel CL (2021) Posterior midline approach for a C2 schwannoma: 2-dimensional operative video. Oper Neurosurg 20(4):E301–E302 10.1093/ons/opaa409 33377140

[2] Eden K (1941) The dumb-bell tumours of the spine. Br J Surg 28(112):549–570

[3] Offiah CE, Day E (2017) The craniocervical junction: embryology, anatomy, biomechanics and imaging in blunt trauma. Insights Imaging 8(1):29–47 27815845 10.1007/s13244-016-0530-5 PMC5265194

[4] Ozawa H, Kokubun S, Aizawa T, Hoshikawa T, Kawahara C (2007) Spinal dumbbell tumors: an analysis of a series of 118 cases. J Neurosurg Spine 7(6):587–593 18074682 10.3171/SPI-07/12/587

[5] Zehri AH, Mugge LA, Kakarla UK, Turner JD, Snyder LA (2025) Management strategies for cervical schwannomas: a comprehensive review. J Neurosurg Spine 42(5):650–658 40053921 10.3171/2024.11.SPINE24802

